#  Hegemonía en el pluralismo médico: articulaciones y relaciones en la atención, un estudio de caso 

**DOI:** 10.1590/0102-311XES092123

**Published:** 2024-04-22

**Authors:** Rocío Santos-Martínez, Elia Nora Arganis Juárez

**Affiliations:** 1 Programa de Maestría y Doctorado en Ciencias Médicas, Odontológicas y de la Salud, Universidad Nacional Autónoma de México, Ciudad de México, México.; 2 Facultad de Medicina, Universidad Nacional Autónoma de México, Ciudad de México, México.

**Keywords:** Antropología de la Salud, Atención Médica, Modelos de Atención de Salud, Ruta Terapéutica, Equivalencia Terapéutica, Medical Anthropology, Medical Care, Healthcare Models, Therapeutic Itinerary, Therapeutic Equivalency, Antropologia da Saúde, Cuidados Médicos, Modelos de Assistência à Saúde, Itinerário Terapêutico, Equivalência Terapêutica

## Abstract

Este estudio tiene como objetivo describir y analizar el pluralismo médico y el tipo de relaciones de hegemonía-subalternidad entre diversas formas o saberes de atención, que se desarrollaron en el itinerario terapéutico de una padeciente de glaucoma, para mostrar el proceso articulatorio y transaccional entre distintos recursos terapéuticos, así como comprender qué elementos estructurales configuraron el itinerario y la elección terapéutica. La investigación es cualitativa, un estudio de caso en el cual se utilizó el enfoque narrativo. Para la reconstrucción de la narrativa se realizó una entrevista semiestructurada, dirigida por una guía temática previamente determinada por un conjunto de categorías apriorísticas, para posteriormente transcribir la entrevista y realizar un proceso de triangulación hermenéutica. Los resultados mostraron, en este caso, que la hegemonía en el pluralismo médico se constituyó mediante relaciones de equivalencia, así, la padeciente sustituyó el uso de medicamentos farmacológicos por terapias de medicina alternativa, no obstante, el proceso relacional de equivalencia se desarrolló en un contexto de significación biomédica, en el cual tratar o controlar la presión intraocular fue la premisa del remplazo. Asimismo, los procesos que desencadenaron la presencia de relaciones hegemónicas se constituyeron por diversos factores sociales, culturales y económicos como el desempleo, la seguridad social y el género, que desempeñaron un papel fundamental durante la búsqueda de la atención y del cuidado.

## Abstract (en)

This study aims to describe and analyze the medical pluralism and the type of hegemony-subordination relation between forms of care or knowledge in the treatment of a patient with glaucoma to show the articulatory and transactional process between several therapeutic resources and understand which structural elements shaped the treatment itinerary and option. This is a qualitative research that used a narrative case study. To reconstruct the narrative, a semi-structured interview was conducted based on a thematic script previously established by a set of a priori categories to later transcribe the data and perform hermeneutic triangulation. Results showed that the hegemony in medical pluralism was based on equivalence relations, so that the patient replaced the use of pharmacological drugs with alternative medicine treatments. However, the relational process of equivalence developed itself in a context of biomedical significance, in which the treatment or control of intraocular pressure configured the substitution premise. Thus, the processes that triggered the hegemonic relations were constituted by various social, cultural, and economic factors such as unemployment, social security, and gender, which played a fundamental role during the search for care.

## Abstract (pt)

Este estudo visa descrever e analisar o pluralismo médico e o tipo de relação de hegemonia-subalternidade entre diversas formas de atendimento ou conhecimentos, que ocorreram no tratamento de um paciente com glaucoma, com a finalidade de mostrar o processo articulatório e transacional entre diferentes recursos terapêuticos, bem como entender quais elementos estruturais moldaram o itinerário e a opção de tratamento. Trata-se de uma pesquisa qualitativa, que utilizou um estudo de caso com abordagem narrativa. Para a reconstrução da narrativa, foi realizada uma entrevista semiestruturada, com base em um roteiro temático previamente estabelecido por um conjunto de categorias *a priori*, para posteriormente transcrever os dados e realizar a triangulação hermenêutica. Os resultados mostraram que a hegemonia no pluralismo médico esteve baseada em relações de equivalência, de modo que o paciente substituiu o uso de medicamentos farmacológicos por tratamentos da medicina alternativa; no entanto, o processo relacional de equivalência desenvolveu-se em um contexto de significância biomédica, na qual o tratamento ou controle da pressão intraocular foi a premissa para a substituição. Desse modo, os processos que desencadearam a presença de relações hegemônicas foram constituídos por fatores sociais, culturais e econômicos diversos como desemprego, previdência social e gênero, os quais tiveram papel fundamental durante a busca por atendimento e cuidado.

## Introducción

En el momento en que los sujetos enferman se origina un conjunto de prácticas y representaciones que pretenden dar resolución a la enfermedad, las cuales se materializan en elecciones y valoraciones terapéuticas de búsqueda y producción del cuidado y atención de la salud ^1^^,^^2^. No obstante, durante este proceso resulta importante destacar que la búsqueda y selección de actividades terapéuticas se entreteje en una compleja realidad social, en la cual las posibilidades de atención son muy variadas y no se restringen al ámbito biomédico ^3^. En este sentido, las acciones enfocadas en la prevención, tratamiento o control de un padecer pueden provenir de distintas formas de atención, las cuales se circunscriben en las diferentes condiciones políticas, religiosas, económicas, étnicas, científicas y técnicas que operan en cada contexto ^4^. A esta coexistencia y utilización de distintos saberes, formas, conocimientos y prácticas de atención-curación se la ha reconocido como pluralismo médico ^5^^,^^6^. Asimismo, es un hecho que los aportes teórico-conceptuales realizados en este ámbito han permitido describir las distintas formas de atención y estrategias terapéuticas utilizadas en diversos padecimientos ^2^^,^^7^^,^^8^, así como desarrollar diferentes posturas analíticas que han podido abstraer y estudiar la realidad del pluralismo médico, a partir de la identificación de ciertos aspectos estructurales de la atención ^9^^,^^10^.

En este aspecto, la Antropología Médica Crítica en América Latina ha estudiado y desarrollado posturas en casi todo el proceso de salud/enfermedad/atención/prevención ^11^ y es en el marco de modelos de atención de la salud, mediante el cual se han construido conceptos que permiten el análisis y estudio del pluralismo médico. Aunado a esto, las corrientes teóricas y metodológicas dominantes de la región han puesto énfasis en analizar a los modelos médicos -y a otros elementos del proceso de salud/enfermedad/atención/prevención- desde una perspectiva que permita articular elementos socioeconómicos y sociopolíticos, que den cuenta de la manera en que estos operan y cambian ^11^.

Algunas de las contribuciones teóricas de la región enfocadas al estudio del pluralismo médico remiten a la construcción conceptual de los modelos médicos: modelo médico hegemónico; modelo médico alternativo subordinado y modelo médico con base en la autoatención ^10^. Desde esta perspectiva, cada modelo es una abstracción de la realidad que representa al conjunto de prácticas y saberes de los curadores, así como de los grupos sociales implicados en el proceso de atención. Los modelos médicos no son entidades que existan plenamente y de manera aislada, por el contrario, se relacionan, articulan, operan y recomponen constantemente en determinados escenarios socioeconómicos y sociopolíticos. Aunque las formas de atención expresadas en estos modelos tienen incompatibilidades y diferencias, en la mayoría de los casos dichas discrepancias pasarán por procesos de complementariedad y no por procesos de completa contradicción. En este sentido, diversos autores han mostrado la manera en que los saberes y las prácticas de los diferentes modelos médicos se retroalimentan con la finalidad de enfrentar y controlar las enfermedades, así como de recuperar el estado de salud ^2^.

Si bien el estudio de los modelos médicos de atención en salud y sus interrelaciones se puede comprender desde diversas perspectivas teóricas, como la expuesta por Janzen ^12^ -enfocada en un análisis sistémico relacional del pluralismo médico, con relaciones simétricas, asimétricas o integradoras-, hasta el momento, uno de los enfoques teóricos más preponderantes en América Latina para estudiar las relaciones de complementariedad entre diferentes formas de atención ha sido el planteamiento de hegemonía-subalternidad. Los procesos relacionales de hegemonía se sustentan en la premisa de que las fuerzas ideológicas, políticas y económicas predominantes en el sistema y en las prácticas médicas no tienden a excluir a otros modelos médicos de atención, sino que a partir de este influjo ideológico del modelo médico hegemónico -formas de atención biomédicas- a otras formas de atención ocurren procesos de subordinación que se manifiestan en apropiación y transformación ^13^. Por lo que, como bien expresa Menéndez ^10^, la hegemonía de las formas de atención biomédicas debe entenderse desde los aportes gramscianos, a partir de los cuales es posible analizar el entrecruzamiento y el establecimiento de relaciones, que conllevan a la instauración de estatutos intelectuales dominantes ^14^.

Pero, más allá de la hegemonía en las formas de atención biomédicas, y de la consecuente subordinación por parte de las otras formas de atención, las relaciones y transacciones que se gestan durante estos procesos deben ser explicadas y analizadas, ya que hasta ahora no han sido explícitamente interpretadas. Para lograr profundizar en el tipo de relaciones y transacciones hegemónicas, que podrían existir entre modelos médicos, los aportes de otras áreas de las ciencias sociales resultan pertinentes para lograr un análisis con mayor profundidad y quizá más exhaustivo.

### Tipo de relaciones y articulaciones hegemónicas entre las formas de atención

Con la finalidad de constituir una propuesta analítica que permita dar una mirada más profunda a la presencia de hegemonía-subalternidad en el pluralismo médico, se retoman los aportes teórico-conceptuales de hegemonía de Laclau & Mouffe ^15^. Desde esta perspectiva, la hegemonía en el pluralismo médico se constituye como una práctica articulatoria, mediante la cual las formas de atención o modelos médicos -entendidos como elementos independientes de una totalidad- se articulan o recomponen con la finalidad de generar negociaciones entre discursos contradictorios ^15^. La característica principal de este proceso articulatorio es que la identidad de cada modelo de atención se verá modificada una vez comenzada la relación, estos cambios en las identidades de los modelos pueden ser explicadas por dos procesos que deben suscitarse: la sobredeterminación o la equivalencia.

#### La sobredeterminación en la hegemonía

Como se ha mencionado, la sobredeterminación es un proceso relacional que puede constituirse debido a que toda identidad social -en este caso las identidades de los modelos médicos o formas de atención- tiene un carácter abierto e incompleto, es decir, ninguna identidad social está completamente constituida, lo cual permite la existencia de articulaciones entre diferentes formas de atención ^15^. Este carácter abierto e incompleto en cada modelo de atención promueve la presencia de la sobredeterminación, entendida como la fusión/combinación de los modelos médicos en el proceso de articulación, en un sentido más práctico, es la presencia de un modelo médico en otro modelo médico ^15^. La sobredeterminación es la relación hegemónica que permite constatar la coexistencia de representaciones y prácticas sociales de un modelo de atención en otro modelo, sin necesidad de que uno a otro se reemplace. No obstante, es un hecho que en algunas ocasiones algunas formas de atención reemplazan a otras, ¿qué sucede con la hegemonía cuando ocurren procesos de reemplazo entre formas de atención?

#### La equivalencia en la hegemonía

Las equivalencias implican un proceso mediante el cual los saberes de diversas formas de atención pueden reemplazarse mutuamente en un mismo contexto de significación ^15^, por ejemplo, el uso de ciertos medicamentos herbolarios y de ciertos fármacos se encuentran en el mismo contexto de significación (a pesar de pertenecer a dos modelos médicos distintos) cuando ambos tienen la misma finalidad terapéutica, por lo que el uso de uno podría reemplazar al otro.

### Una propuesta de análisis a las relaciones hegemónicas en el pluralismo médico

Mediante la propuesta teórico-conceptual de Laclau & Mouffe ^15^ es posible identificar que la hegemonía se puede constituir por relaciones de sobredeterminación y equivalencia, las cuales se caracterizan por tener prácticas articulatorias.

Una vez explicadas las principales características de las relaciones hegemónicas, la propuesta analítica radica en identificar a través del pluralismo médico -el cual puede expresarse mediante itinerarios terapéuticos u otras herramientas teórico-metodológicas- los siguientes tipos y grados de relación entre las formas de atención:

(i) Sobredeterminación/Acuerdos parciales;

(ii) Equivalencia: reemplazo en contextos de significación similares.

Para lograr conceptualizar la presencia de hegemonía y subalternidad en esta propuesta de análisis, se debe inferir que la búsqueda de atención y la utilización de diversas estrategias terapéuticas se circunscriben en contextos culturales e institucionales regulados políticamente, en el cual existen jerarquías y hegemonías ^15^^,^^16^. Desde este panorama, la propuesta expuesta posibilita identificar de manera exhaustiva el tipo de relaciones que se desarrollan en construcciones de hegemonía/subalternidad, que den cuenta de manera más precisa de la multiplicidad y diversidad de las formas de atención utilizadas por los sujetos que padecen.

Ante lo anteriormente mencionado, el objetivo de este trabajo es ejemplificar esta propuesta de análisis a las relaciones hegemónicas, mediante el estudio de caso de un itinerario terapéutico de una persona con glaucoma. El estudio de caso expuesto permite ilustrar cómo la búsqueda y elección de atención se circunscriben en constantes relaciones de hegemonía, configuradas por diversos factores sociales, culturales o económicos.

## Contexto de la investigación y del estudio de caso

La información etnográfica que se presenta deriva de una investigación sobre itinerarios terapéuticos en personas con glaucoma, la cual se desarrolló en la Ciudad de México (México) durante el año de 2022. El estudio principal se centró en describir y analizar la configuración de los itinerarios terapéuticos, a partir de la construcción sociocultural del padecer y las interrelaciones de los diferentes saberes médicos que se presentan durante el desarrollo del itinerario.

La investigación se realizó desde un enfoque narrativo, la finalidad fue retomar la experiencia de los sujetos y conocer e identificar el conjunto de saberes -entendidos como representaciones y prácticas-, las formas de institucionalización y el contexto de producción de la narrativa ^16^. En el estudio colaboraron siete sujetos con diagnóstico biomédico de glaucoma, de los cuales cuatro fueron hombres y tres fueron mujeres. Los participantes fueron contactados de manera directa por medio de redes sociales de internet, en grupos de ayuda y difusión de información para personas con glaucoma, todos los interesados en contribuir aceptaron su participación mediante un consentimiento informado y el acuerdo de mantener su anonimato y la confidencialidad de su información, por lo que los nombres de cada colaborador fueron modificados. Para la reconstrucción de las narrativas se utilizó la entrevista semiestructurada, dirigida por una guía temática previamente determinada por un conjunto de categorías apriorísticas ^17^.

A cada colaborador residente de la Ciudad de México se lo entrevistó de manera presencial, mientras que para los participantes de otras ciudades se utilizaron aplicaciones digitales de videollamada como Google Meet (https://meet.google.com/) o Zoom (https://zoom.us/). Cada participante fue entrevistado de tres a cuatro veces con una duración de dos horas cada sesión.

El estudio de caso presentado en este trabajo corresponde a la colaboradora Mariana Sandoval, de 62 años, originaria de la ciudad de Mérida, en el estado de Yucatán (México), con actual residencia en ciudad Caucel, la cual es considerada una zona satélite de la ciudad de Mérida. En el momento en que se realizaron las sesiones de entrevista, Mariana estudiaba cinematografía y trabajaba en la escuela a la que asistía, ya que de esta manera retribuía las clases que le eran proporcionadas. La colaboradora indicó haber estudiado enfermería y desempeñado varios cargos de coordinación en instituciones biomédicas, no obstante, con la presencia asidua de malestares como dolor de cabeza, decidió ejercer laboralmente en otras áreas, por lo que estudia y posteriormente se dedica a la repostería. Una vez detectado y diagnosticado el glaucoma, ella comenzó a estudiar cinematografía. Mariana recibe el diagnóstico biomédico de glaucoma durante el mes de octubre del 2020, por lo que la búsqueda de atención se suscita en un contexto de pandemia, cierre de servicios de salud (y de otro tipo de servicios), además de reconversión de hospitales y clínicas a centros de atención exclusiva para COVID-19.

## Materiales y métodos

El presente escrito es un estudio de caso que dimana de una investigación enfocada al análisis de itinerarios terapéuticos de personas con glaucoma, la cual fue sometida al Comité de Ética e Investigación del Programa de Maestría y Doctorado de Ciencias Médicas, Odontológicas y de la Salud de la Universidad Nacional Autónoma de México (código de aprobación PMDCMOS/CE5/2023).

El estudio de caso pretende ejemplificar y analizar el tipo de relaciones hegemónicas que se desarrollan entre las diferentes formas de atención, con base en la propuesta teórico-conceptual anteriormente descrita.

Para realizar el análisis se indagó el proceso de búsqueda de atención de Mariana mediante la reconstrucción del itinerario terapéutico. El itinerario tuvo como premisa recoger la experiencia, desentrañar el proceso dinámico de búsqueda de atención y el contexto en dónde la colaboradora habitaba ^7^. La reconstrucción del itinerario se realizó mediante entrevistas semiestructuradas, determinadas por un conjunto de categorías y subcategorías apriorísticas enfocadas en el uso de diferentes modelos médicos y en los elementos contextuales, que determinaron la elección terapéutica (Cuadro 1).

**Cuadro 1:** Categorías y subcategorías apriorísticas para la entrevista semiestructurada del estudio de caso.

CATEGORÍAS	SUBCATEGORÍAS
Modelo médico hegemónico	Tipo de atención utilizados Recursos terapéuticos utilizados Representaciones sociales sobre la efectividad
Modelo médico alternativo subordinado	Tipo de atención utilizados Recursos terapéuticos utilizados Representaciones sociales sobre la efectividad
Modelo médico de autoatención	Recursos terapéuticos utilizados Representaciones sociales sobre la efectividad
Elementos contextuales de elección terapéutica	Condiciones sociales, culturales y económicas Acceso a servicios de salud

Fuente: elaboración propia con base en Menéndez ^10^ y Cisterna ^17^.

Las entrevistas se realizaron los fines de semana de los meses de septiembre, octubre y noviembre del 2022 en cuatro sesiones con una duración de una a dos horas por fase, cada entrevista fue realizada en la plataforma digital Google Meet y audio-grabada previo consentimiento informado. Para el análisis de los datos se transcribió la entrevista literalmente, después se llevó a cabo la construcción de la narrativa y un proceso de triangulación hermenéutica, el cual consistió en reunir y cruzar la información mediante los siguientes pasos:

(i) Se seleccionó información de acuerdo con los siguientes criterios: pertinencia con las categorías-subcategorías y relevancia por recurrencia o asertividad;

(ii) Una vez seleccionada la información, se triangularon los datos, a través de la elaboración de conclusiones ascendentes, es decir, conclusiones por subcategorías o conclusiones de primer nivel, para posteriormente agrupar las conclusiones subcategoriales en las categorías pertinentes y conformar conclusiones por categorías o de segundo nivel.

## Resultados

### Antecedentes de la enfermedad

Mariana recibió un diagnóstico biomédico de ateromatosis bilateral cerebral en el 2018, dos años antes de recibir el de glaucoma, por lo que los dolores de cabeza eran situaciones constantes durante los últimos cuatro años y el consumo de medicamentos (antihipertensivos y anticoagulantes) también era habitual.

### El itinerario terapéutico de Mariana

Cuando aparecen los malestares visuales inicia la búsqueda de atención, en este itinerario terapéutico se realizan prácticas de autoatención y se recurre a la atención biomédica privada e institucional, como se ilustra en el Cuadro 2.

**Cuadro 2:** Prácticas de atención en el itinerario terapéutico de Mariana.

1. Automedicación con analgésicos	2. Asistencia a consultorios adyacentes a farmacias (ausencia de resolución al padecimiento)	3. Servicios de salud públicos de la Secretaría de Salud de Yucatán, México (ausencia de resolución al padecimiento por reconversión hospitalaria a servicios de atención para COVID-19)	4. Atención biomédica privada en establecimientos especializados en oftalmología	5. Establecimiento de un diagnóstico biomédico y comienzo de tratamiento (seguimiento de tratamiento biomédico por seis meses)
6. Abandono de tratamiento biomédico por situaciones financieras	7. Aplicación (mediada por la red de apoyo familiar) de gotas provenientes de la herbolaria, para control del glaucoma	8. Uso de gotas provenientes de la herbolaria por dos meses	9. Abandono de la terapia alternativa por situaciones financieras	10. Acciones de autocuidado para aminorar los síntomas

Fuente: trabajo de campo, 2022.

### Modelo médico hegemónico - uso de formas de atención biomédicas

Las formas de atención biomédicas iniciaron una vez que se percibió un dolor de cabeza incesante y una hemorragia subconjuntival. Los tipos de atención utilizados correspondientes a la forma de atención biomédica son los públicos y privados. Durante el transcurso del itinerario terapéutico se pudo mostrar que la primera instancia de búsqueda biomédica fueron los consultorios adyacentes a farmacias, en los cuales la falta de una explicación diagnóstica y una adecuada resolución promovieron que la colaboradora buscara atención en servicios de atención públicos de la Secretaría de Salud de Yucatán. Estos brindan atención a personas sin seguridad social y sin capacidad de pago, sin embargo, por situación pandémica, Mariana no pudo ser tratada ni canalizada a algún otro sitio, lo que derivó en la búsqueda de atención médica privada especializada, en la cual encontró atención y explicación a su padecer. Los recursos terapéuticos biomédicos utilizados para tratar el glaucoma se concretaron principalmente en dos categorías:

(i) Recursos farmacológicos: medicamento compuesto por dorzolamida, timolol y brimonidina (destinados al control de la presión intraocular), además de diurético oral;

(ii) Recursos quirúrgicos: iridectomía (procedimiento para extirpación parcial del iris) en ambos ojos y trabeculectomía (creación de una pequeña apertura en la esclerótica) en ojo izquierdo.

### Modelo médico de autoatención

La autoatención representa el punto de partida del itinerario, además de constituirse como un proceso constante durante el mismo. Una vez que Mariana comenzó a percibir sensaciones de dolor y malestar, se suscitaron acciones enfocadas a la autoatención, mediante la autoadministración de analgésicos, cuando el uso de estos medicamentos no controló el síntoma percibido, se decidió detener el consumo y acudir a la forma de atención biomédica. Posterior a ese momento, la autoatención no se detiene, ya que otros factores como la efectividad percibida en el tratamiento indicado por el curador o médico, aspectos económicos o incluso elementos relacionados con el tiempo destinado al uso de la terapéutica, desencadenaron acciones o procesos de autoatención, como fueron el cese o la modificación de la terapéutica indicada. Los dos aspectos expuestos se muestran en los siguientes testimonios de Mariana:

“*No se me pasaba el dolor y decía ‘qué raro, si ni el tramadol me respetó el dolor aquí hay algo que no está lógico, hay algo que está incongruente’, mi familia se da cuenta de que yo no me sentía bien porque me quise parar y no me pude parar en ese momento, es cuando mi hija mayor y mi esposo deciden llevarme al médico*”.

“*Fue muy difícil llevar el tratamiento a nivel económico, a nivel físico fue bastante engorroso tener que ponerte las gotas tales cada dos horas, las gotas tales cada cuatro horas, tomarte una pastilla cada tantas horas, yo tenía una pizarra aquí en casa y tenía montada la pizarra literal, pero a nivel económico si fue muy difícil para mí, ya que los medicamentos son sumamente caros, son medicamentos en los cuales debes pagar 1200 pesos* [mexicanos] *por gotas que te van a durar 15 días, ahora sí que es bastante difícil, bastante engorroso, realmente al final de cuentas terminas haciendo, lo que yo he hecho, dejando el tratamiento y saber que va a pasar y mientras avanza esta situación, aun sabiendo que es irreversible*”.

Después del diagnóstico biomédico de glaucoma transcurrieron seis meses de constante revisión biomédica, sin embargo, ante el incesante gasto destinado a la atención y las repercusiones financieras que esto conllevó, Mariana decidió comenzar a utilizar formas de atención alternativas -herbolaria-, por lo que sustituyó el uso del medicamento oftalmológico por gotas de CBD (compuesto químico derivado de la planta *Cannabis sativa*), las cuales fueron recomendadas por familiares y provistas por su hija menor, por lo que la prescripción no fue guiada por un curador especializado. La efectividad de estas formas de atención, a diferencia de las biomédicas, no dependió de la rapidez con que se eliminaron los síntomas o si hubo un proceso de explicación que permitiera al sujeto comprender el origen de la enfermedad o el mecanismo de acción de la terapia, en este caso, se limitó al hecho de eliminar o aminorar los síntomas percibidos y sentidos, lo siguiente se ilustra en la aseveración de Mariana:

“*¿Cuál fue el cambio?, el cambio que yo sentí cuando consumí las gotas de CBD, no fumaba ni me hacía mis tés de las hojitas, aclaro, me tomaba 10 gotas diario y sentía menos presión en el ojo, sí sentía menos presión en el ojo definitivamente, mentiría si dijera que estaba toda relajada, eso no es cierto, pero sí había una sensación de que en el ojo no había tanta presión, no cambió la visión, pero esa sensación de pesadez de ojo era muchísimo más ligera, esa sensación definitivamente y cierto grado de relax así bonito*”.

La efectividad de la autoatención se reflejó en el control de los síntomas percibidos y sentidos, ya que si la autoadministración de algún medicamento no eliminaba el dolor o la sensación de malestar se percibía como poco efectivo y se recurría a otras formas de atención. Pero esto no solo ocurrió con medicamentos de la biomedicina, ya que el inicio y cese de terapéuticas de otras formas de atención también fueron relativamente autónomas.

La autoatención de este itinerario comprendió dos momentos:

(i) La automedicación con el suministro de analgésicos como tramadol para control de dolor de cabeza o la toma de gotas de CBD para control del glaucoma;

(ii) El autocuidado caracterizado por acciones de prevención y atención a nivel individual, como lo fue la realización de acondicionamiento físico tipo calistenia. Las actividades enfocadas al autocuidado fueron percibidas como efectivas, ya que se destinaron a la atención de otros factores relacionados con el sentir y las emociones provocadas por el padecimiento y no solamente a elementos de control de síntomas.

## Discusión y conclusiones

Por lo que se ha planteado en este estudio de caso, el tipo y grado de relación hegemónica que se presentó fue el de equivalencia, ya que una vez que se tuvo un diagnóstico y tratamiento, diversos aspectos sociales, económicos y culturales condicionaron el reemplazo de esta forma de atención por otra que fuera más conveniente y módica. Durante este proceso de equivalencia, la autoatención actuó como punto nodal de la articulación, ya que el reemplazo del medicamento oftalmológico por gotas de CBD fue incentivado por la red familiar de apoyo de Mariana, por lo que la prescripción, supervisión y cese de esta forma de atención no fue controlada por un curador.

De igual modo, otros estudios han reportado importantes hallazgos respecto a la presencia y uso de diferentes formas de atención en itinerarios terapéuticos o trayectorias de atención, como el efectuado en el estado de Chiapas (México) ^18^, el cual mostró cómo, durante la búsqueda de atención médica, de niños con diagnóstico biomédico de leucemia, se consultaron y utilizaron diferentes formas de atención como la medicina naturista o tradicional, además se expuso la importancia de la autoatención como medida de resolución a síntomas por parte de los cuidadores. Asimismo, en Argentina ^19^ se analizaron las trayectorias de atención de personas con enfermedades crónicas no transmisibles, en este estudio se constató el uso y la efectividad percibida de los modelos médicos por parte de los participantes, el análisis demostró que la articulación de saberes y la producción de acciones conjuntas, derivadas de esta articulación, mejoraron los procesos de atención.

Otro aporte académico realizado en México ^20^, específicamente en zonas mayahablantes, reveló que usuarios que asisten a centros de salud de atención primaria para control y tratamiento de diabetes tipo 2 utilizaron diferentes formas de atención, por ejemplo, la medicina alternativa fue usada en algunos casos como complemento y en otras situaciones sustituyó el uso de la medicina biomédica, con esta ejemplificación y con los aportes teóricos mostrados en el presente estudio de caso, se podrían retomar dichos resultados y demostrar que las acciones de complementar y sustituir se equiparan a las relaciones hegemónicas de sobredeterminación y equivalencia respectivamente.

Asimismo, otros autores ya han referido procesos de articulación en el pluralismo médico o terapéutico y lo han denominado complementariedad o síntesis terapéutica ^21^. Los términos aluden a la combinación de diferentes saberes médicos para afrontar un mismo padecimiento ^21^. En este sentido, estos conceptos posibilitan comprender que ya se han analizado y conceptualizado relaciones articulatorias entre representaciones y prácticas de diferentes formas de atención, las cuales son comparables con las relaciones hegemónicas de sobredeterminación expuestas en este escrito.

Aunado a esto, al igual que en este estudio de caso, la investigación de México ^20^ mostró que la autoatención desempeñó un papel sumamente importante durante los procesos relacionales, ya que la utilización de saberes alternativos fue recomendada mayormente por la red social de apoyo, por lo que la autoatención fue el punto nodal mediante el cual se articularon y relacionaron diferentes formas de atención.

Además de comprender los procesos que desencadenan la presencia de relaciones hegemónicas, el estudio de caso presentado permitió entender que dichos procesos se constituyeron por diversos factores sociales, culturales y económicos, situaciones que se han analizado en múltiples investigaciones, que muestran la complejidad de las interacciones entre lo individual y lo colectivo al tratar de recuperar la salud en contextos diferenciados ^22^^,^^23^^,^^24^.

Conocer y comprender los elementos estructurales que configuran de manera general al itinerario, permite demostrar que los sujetos y los grupos sociales buscarán y utilizarán cualquier forma de atención que esté a su alcance y se adapte a sus condiciones ^20^. En este sentido, se pudo certificar, que en el caso de Mariana, situaciones como el desempleo no le permitieron tener acceso a prestaciones de seguridad social y salud (característica importante en el sistema de salud mexicano, para poder acceder a servicios de salud públicos), por lo que la búsqueda de atención se desarrolló como un proceso complicado y largo, ya que sus posibilidades de atención se redujeron a los servicios de atención privados y a los servicios de atención públicos para población sin seguridad social. Estos últimos poco accesibles por el momento en el cual ella fue diagnosticada con glaucoma (etapa de reconversión hospitalaria por pandemia de COVID-19), además, aun con la provisión de consulta médica especializada, el suministro de medicamentos o la realización de procedimientos quirúrgicos resultaron ser elementos difíciles de conseguir, si no se tiene acceso a servicios de salud por seguridad social o asequibilidad financiera.

Asimismo, factores como el estado civil repercutieron en la posibilidad de acceder a seguridad social y servicios de salud, como se demuestra en la siguiente aserción:

“*Lamentablemente, no tenía, de hecho, hasta la fecha, hasta el día de hoy, no tengo seguridad social, mi pareja si tiene seguridad social y pues ya estamos viendo la necesidad de que él me pueda afiliar como concubina, soy concubina con una promesa en junio, como actualmente las leyes ya cambiaron, las concubinas tenemos el mismo derecho que las casadas legalmente, pero para algunos trámites el bendito papelito confirma*”.

Otro aspecto por destacar es que las representaciones sociales y significados atribuidos a la maternidad, así como las normas de género enmarcadas en el rol femenino y de madre hicieron que la atención no fuera inmediata, ya que se antepusieron significaciones de cuidado familiar, por lo que la búsqueda de atención ocurrió hasta que los síntomas se acrecentaron.

“*Llegué a la casa, seguí tomando el antihipertensivo y mi hija tenía cita con la psiquiatra en una clínica particular, en ese momento me habló la chica para corroborarme la cita y le dije sabes no voy a poder asistir a esa cita médica, porque la que necesita cita médica soy yo, de hecho estoy buscando un oftalmólogo, porque tengo un problema en uno de los ojos y me dijo pues aquí tenemos una oftalmóloga, si gusta le hago la cita con ella, entonces el dinero que yo iba a usar para la consulta de mi hija lo usé para mi consulta. Mira, soy el pilar de la familia, soy la matriarca por excelencia, soy la que mueve los hilos de esta familia, hubo un ligero caos porque nadie sabía qué hacer, nadie sabía cómo manejar la situación*”.

Además, se observaron otras problemáticas no solamente asociadas a factores económicos para adquirir los medicamentos. La prescripción indicada por los médicos también representó un elemento que influyó en el proceso articulatorio entre formas de atención, debido a que se concibieron representaciones sociales sobre restricciones en las actividades cotidianas, derivadas de la frecuente aplicación de medicamentos de la biomedicina.

“*Cuando yo pruebo el tomar por las noches las gotas de CBD se me hizo menos engorroso*”.

En conclusión, la posibilidad de retomar aportes teóricos-conceptuales de las ciencias sociales permite comprender con mayor profundidad la manera en que ocurren las relaciones o articulaciones entre formas de atención.

En el estudio de caso de Mariana, se puede corroborar que diferentes factores estructurales determinaron la presencia de relaciones hegemónicas entre los modelos médicos de atención, en este aspecto, se desarrolló una relación hegemónica de equivalencia entre la aplicación de gotas oftálmicas para la disminución de la presión intraocular y entre las gotas de CBD. La relación se circunscribió en una lógica de equivalencia caracterizada por la presencia de una fuerza hegemónica del modelo biomédico, ya que la búsqueda de un elemento de reemplazo se definió en un contexto de significación biomédico, en el cual tratar o controlar la presión intraocular fue la premisa del reemplazo. Un análisis más exhaustivo a la presencia de hegemonía permite entender que todos los modelos médicos de atención desempeñan un papel importante durante la búsqueda de atención y producción del cuidado de la salud, ya que cada forma de atención se une o complementa para mejorar los procesos de atención o subsanar las limitaciones, que cada modelo médico pudiera presentar.
